# Biomarkers and Future Perspectives for Hepatocellular Carcinoma Immunotherapy

**DOI:** 10.3389/fonc.2021.716844

**Published:** 2021-09-06

**Authors:** Yuqing He, Mengyao Lu, Jing Che, Qian Chu, Peng Zhang, Yuan Chen

**Affiliations:** ^1^Department of Oncology, Tongji Hospital, Tongji Medical College, Huazhong University of Science and Technology, Wuhan, China; ^2^College of Life Sciences, Wuhan University, Wuhan, China

**Keywords:** hepatocellular carcinoma, biomarkers, immune checkpoint inhibitors, immune-related adverse events, immunotherapy

## Abstract

Hepatocellular cancer is the sixth most frequently diagnosed malignant disease worldwide, and was responsible for tens of millions of deaths in 2020; however, treatment options for patients with advanced hepatocellular carcinoma remain limited. Immunotherapy has undergone rapid development over recent years, especially in the field of immune checkpoint inhibitors (ICIs). These drugs aim to activate and enhance antitumor immunity and represent a new prospect for the treatment of patients with advanced cancer. Nevertheless, only a small proportion of liver cancer patients currently benefit from ICI-based treatment, highlighting the need to better understand how ICIs and tumors interact, as well as identify predictive biomarkers for immunotherapeutic responses. In this review, we highlight clinical trials and basic research in hepatocellular carcinoma, with a particular focus on predictive biomarkers for the therapeutic efficacy of ICIs. Predictive biomarkers for immune-related adverse events are also discussed.

## Introduction

Hepatocellular carcinoma (HCC) accounts for over 80% of all primary liver malignancies, while liver cancer ranked as the sixth most frequently diagnosed cancer in 2020, resulting in 83 million deaths (1). Despite these statistics, HCC treatment remains a major healthcare challenge globally. Additionally, because symptoms and physical characteristics of HCC are not easily detected, 80% of patients diagnosed with HCC miss out on curative treatment (2).

HCC typically develops in a background of underlying inflammatory liver disease, especially that associated with hepatitis B (HBV) or hepatitis C virus (HCV) infection (3), while nonalcoholic fatty liver disease (NAFLD) is rapidly becoming a key etiological factor for HCC in many Western countries (4, 5). Current treatment modalities for patients with nonadvanced HCC include resection, transplantation, ablation, or chemoembolization, while patients with advanced HCC receive systemic treatment (6). However, progress in the development of treatments for advanced HCC has been limited, partly due to the complex and heterogeneous etiology of this disease. Additionally, the most common driver mutations (*TERT* promoter, *CTNNB1*, *TP53*, and *ARID1A* mutations) have not yet been shown to be suitable therapeutic targets (7). Although first-line multikinase inhibitors (sorafenib and lenvatinib) can prolong the survival of patients with advanced HCC (8–10), and multitarget tyrosine inhibitors (e.g., regorafenib and cabozantinib) and vascular endothelial growth factor (VEGF) receptor inhibitors (e.g., ramucirumab) can provide benefit for patients who previously tolerated sorafenib (11), most cases of HCC show tolerance or become refractory to these agents during the clinical course of the disease (12). Accordingly, the median overall survival (OS) for patients treated with these agents remains under 15 months.

Cancer immunotherapy has undergone rapid development in recent years, especially in the field of immune checkpoint inhibitors (ICI). Immune checkpoint-related molecules, such as programmed cell death-1 (PD-1), cytotoxic T-lymphocyte-associated antigen 4 (CTLA-4), T-cell immunoglobulin, mucin domain-3 (TIM-3), and lymphocyte activating-3 (LAG-3), are important components of the negative feedback regulatory mechanism that serves to suppress excessive immune responses. They are constitutively upregulated in various tumors, generating T-cell exhaustion or anergy, and thereby helping tumors evade immune surveillance (13). The rationale behind utilizing ICIs is to restore and enhance antitumor immunity by relieving the immunosuppressive effects of immune checkpoint-related molecules. The development of anti-PD-1, anti-programmed cell death-ligand 1 (PD-L1), and anti-CTLA-4 monoclonal antibodies has advanced the treatment for advanced cancer, resulting in numerous attempts to apply ICIs for the treatment of multiple advanced solid malignancies, including HCC.

Based on encouraging results from the CheckMate 040 and KEYNOTE-224 clinical trials, the United States Federal Drug Administration (FDA) has granted accelerated approval for the PD-1 inhibitors nivolumab and pembrolizumab as second-line treatments for advanced HCC. The CheckMate 040 phase I/II trial obtained objective response rates (ORRs) of 15% (dose-escalation phase) and 20% (dose-expansion phase) in patients treated with nivolumab (14). Meanwhile, the KEYNOTE-224 phase II trial reported an ORR of 17% for pembrolizumab monotherapy for HCC patients previously treated with sorafenib (15). Disappointingly, however, both the CheckMate 459 and KEYNOTE-240 phase III trials, which evaluated nivolumab *versus* sorafenib and pembrolizumab *versus* best supportive care, respectively, failed to meet their predetermined primary endpoints of OS (16, 17). Overall, ICI monotherapy has shown limited efficacy in HCC, benefiting only a limited subgroup of patients. More results of clinical trials for ICIs in HCC are summarized in **Table 1**.

**Table 1:** Clinical trials of immune-checkpoint inhibitors in hepatocellular carcinoma.

Study Name				PFS	OS		Estimated
(Reference)	Agent	ORR (%)	DCR (%)	(median, months)	(median, months)	Phase	Enrolment (*n*)
CheckMate 040 (14)	Nivolumab	19.6	64.5	4.0	15.0	I/II	214
CheckMate 040; Asian cohort analysis (18)	Nivolumab	15.2	49.4	NA	14.9	I/II	85
NCT01693562 (19)	Durvalumab	10.3	33.3	NA	13.2	I/II	39
NCT01008358 (20)	Tremelimumab	17.6	76.4	6.5	8.2	II	20
NCT02658019 (21)	Pembrolizumab	32.1	46.4	4.5	13.0	II	28
Keynote-224 (15)	Pembrolizumab	17.3	61.5	4.9	12.9	II	104
CheckMate 459 (17)	Nivolumab *vs.* sorafenib	15.4 *vs.* 7.0	NA	3.7 *vs.* 3.8	16.4 *vs.* 14.7	III	743 (371 *vs.* 372)
Keynote-240 (16)	Pembrolizumab *vs.* placebo	18.3 *vs.* 4.0	62.2 *vs.* 53.3	3.0 *vs.* 2.8	13.9 *vs.* 10.6	III	413 (278 *vs.* 135)

ORR, overall response rate; DCR, disease control rate; OS, overall survival; PFS, progression-free survival; NA, not available.

In contrast, the results from the IMbrave150 phase III trial have been encouraging (22). This trial enrolled 501 patients with advanced HCC who had not previously received systemic treatment, two-thirds of whom received atezolizumab (anti-PD-L1 blockade) plus bevacizumab (VEGF monoclonal antibody), while the others received sorafenib monotherapy. The median progression-free survival (PFS) was 6.8 months in the combination arm and 4.3 months in the sorafenib group. In addition, OS at 12 months was 67.2% with atezolizumab + bevacizumab and 54.6% with sorafenib. The two subgroups displayed similar toxic effects, with an incidence of 56.5% for grade 3 or 4 adverse events in the combination arm and 55.1% in the sorafenib arm. Of note, these data are momentous, as they identify the first therapy to improve OS and PFS beyond the standard of care sorafenib in treatment-naïve patients (23). After more than a decade of stagnation in the treatment of advanced HCC, these therapeutic strategies changed the *status quo* and have entered into clinical practice. The mechanisms underlying the effects of ICI/anti-VEGF-agent combination treatment have also been elucidated, and have been reviewed in detail (24, 25).

Even though the prospect for ICIs seems to be excellent, numerous difficulties remain to be resolved. Pivotal among these is the low response rate in patients treated with ICI monotherapy, with treatment benefiting only 15%–20% of patients with advanced HCC (14, 15). The incidence of immune-related adverse events (irAEs) is another important concern. A better understanding of the dialog between ICIs and tumors is essential, as is the identification of predictive biomarkers for treatment response and toxicity. In this review, we focus on clinical trials and basic research in HCC, with particular emphasis on predictive biomarkers for the therapeutic efficacy of ICIs. Predictive biomarkers for irAEs are also discussed.

## Potential Predictive Biomarkers for ICI-Based Treatment

**Figure 1:**
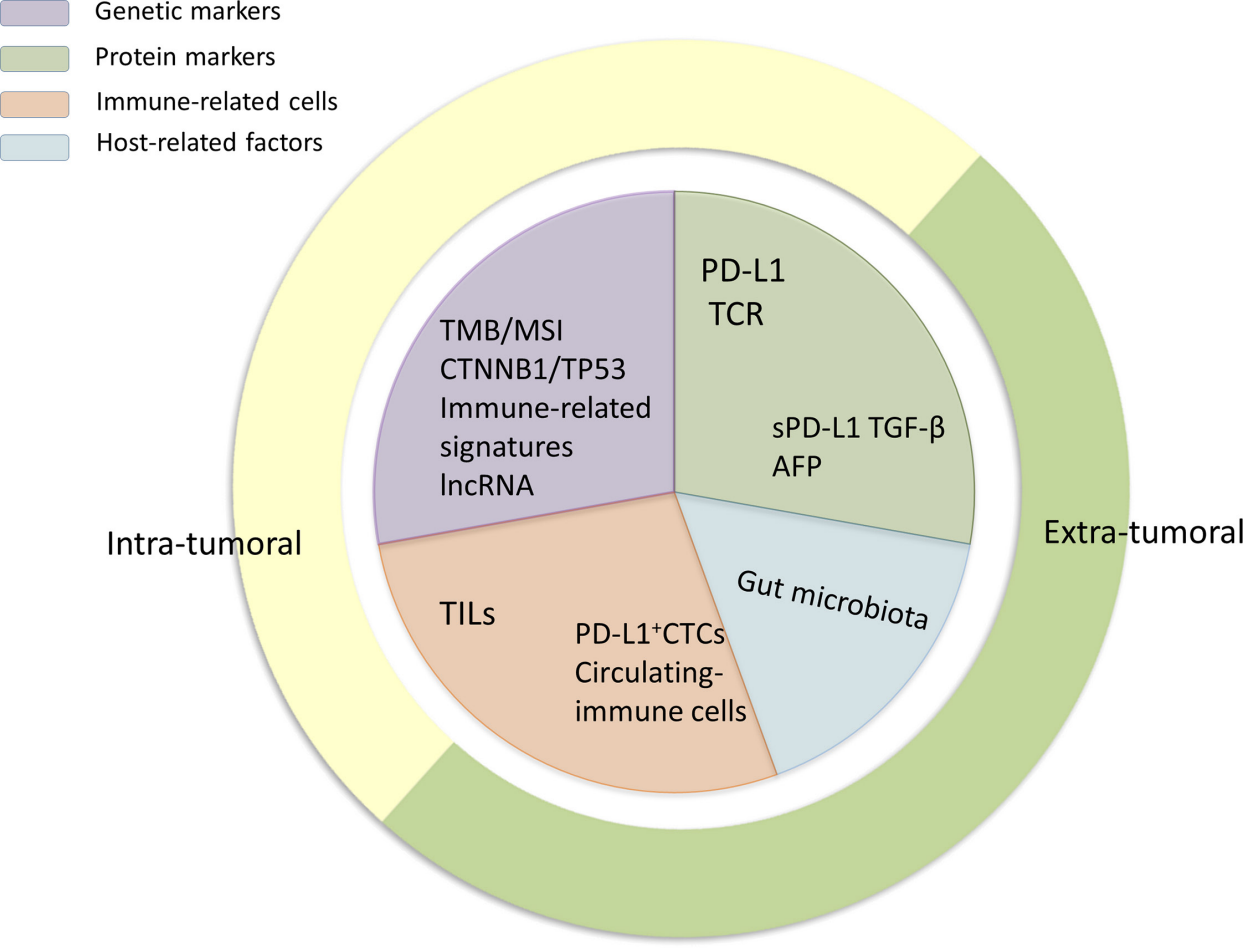
The classification of biomarkers. The biomarkers introduced in this review can be divided into protein markers, gene markers, immune-related cells, and host-related cells. Moreover, according to their localization *in vivo*, they can be further subdivided into intratumoral and extratumoral biomarkers as they are detected using different methods.

**Figure 2:**
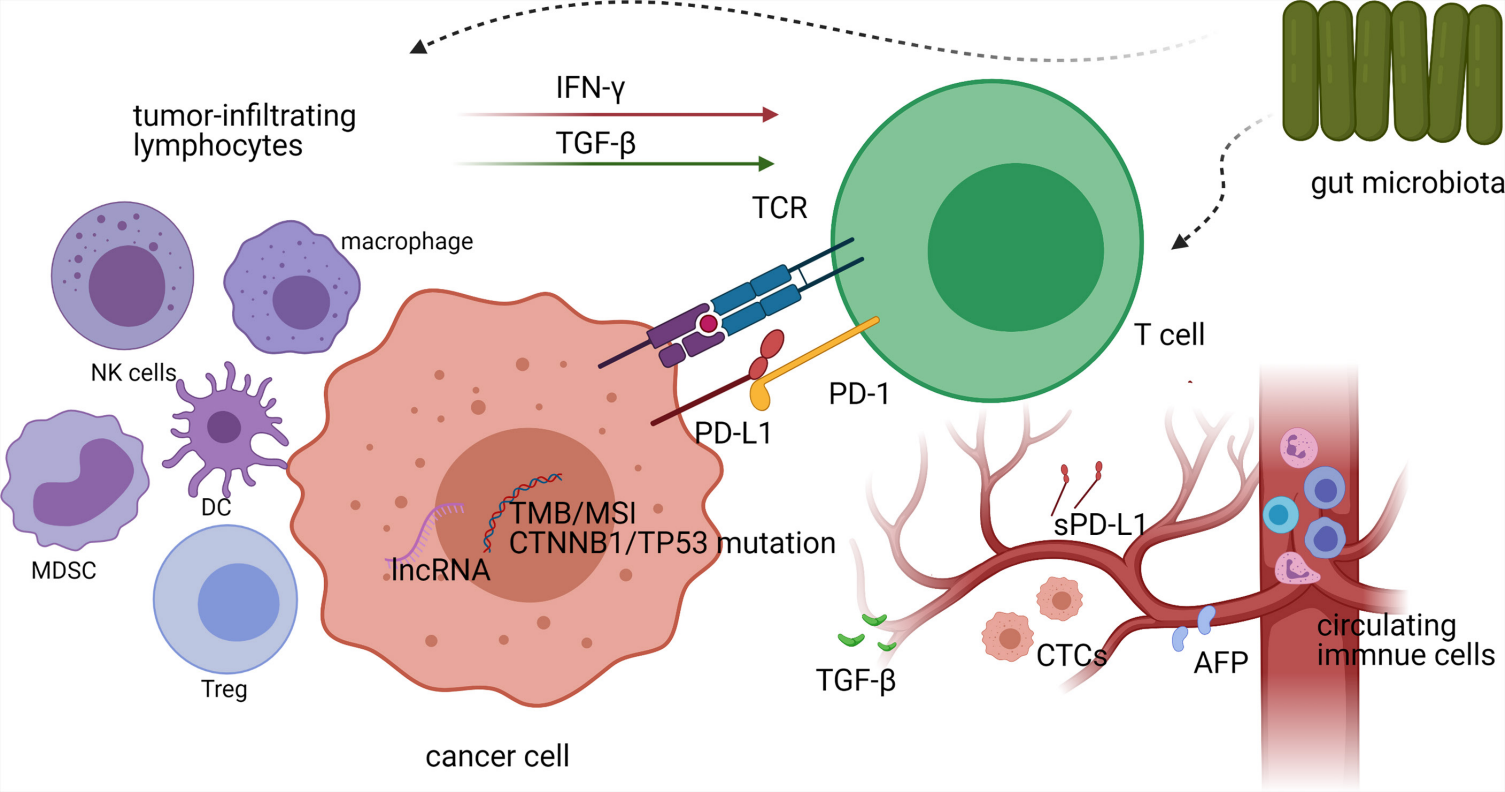
A schematic representation of biomarkers for predicting treatment responses to immune checkpoint inhibitors (ICIs) *in vivo* and their interactions with each other. Both the genetic characteristics and the expression of PD-L1 in tumor cells, as well as the density and diversity of tumor-infiltrating CD8^+^ T cells, have the potential to predict the efficacy of ICI treatment in hepatocellular carcinoma (HCC). Other tumor-infiltrating lymphocytes in the tumor microenvironment affect T-cell activity by secreting cytokines (such as IFN-γ and TGF-β), thereby affecting the efficacy of ICI treatment. The gut microbiota also affects the responses to ICI treatment by influencing the activity of tumor-infiltrating lymphocytes (TILs), while circulating biomarkers in peripheral blood can also be used as predictors of immunotherapeutic efficacy.

Because immunotherapy for HCC is still in its infancy, studies relating to predictive biomarkers for ICI treatment response are scarce. Nevertheless, several valuable data on potential biomarkers have emerged in recent years, including genetic and protein markers, immune-related cells, and host-related factors (**Figures 1**, **2**), which are described below.

### PD-L1 Expression

PD-L1 is dynamically and widely expressed on the surface of tumor cells, antigen-presenting cells, and other immune cells. PD-L1 expression is reported to be generally low in HCC (~10% of tumor cells) (26) and is associated with recurrence and shorter OS (27). In addition, high PD-L1 expression in inflammatory cells within the tumor microenvironment (TME) correlates with high serum alpha-fetoprotein (AFP) levels, macrovascular invasion, and poor differentiation, resulting in increased tumor aggressiveness (28). PD-L1 was the first proposed predictive biomarker for responses to ICIs, and the relationship between PD-L1 expression and ICI treatment response has been extensively explored in HCC-related clinical trials.

Several results of clinical trials in which PD-L1 was evaluated as a predictive biomarker for ICI responses have been published (the data are summarized in detail in **Table 2**). In the CheckMate 459 phase III trial, although nivolumab did not elicit prominent improvements in OS as a first-line treatment for HCC, PD-L1-positive tumors nevertheless showed a better response to nivolumab compared to sorafenib (17). This agreed with the results of the KEYNOTE-224 trial, which showed that PD-L1 expression was associated with the response to pembrolizumab, even though the results were not statistically significant (15). However, the predictive value of PD-L1 remains unclear. Disappointing response rates were consistent across all patients in both the CheckMate 040 and NCT02658019 trials, regardless of PD-L1 expression levels (14, 18, 21). Even so, further analysis of patients in the CheckMate 040 trial showed that tumor PD-L1 expression was associated with improved OS, although objective responses could still be observed in PD-L1-negative patients treated with nivolumab. These observations highlight that PD-L1 expression alone may not serve as an adequate biomarker for responses to ICI treatment (29). Although these trials provide valuable clinical data that allow the evaluation of the predictive effect of PD-L1 expression, they have their limitations. First, these are retrospective analyses with small sample sizes. Secondly, there are limitations associated with the detection of PD-L1 expression (discussed in detail later), as well as inconsistencies in sample sources; although they are all collected at baseline, some samples are fresh and some are archival.

**Table 2:** Predictive biomarkers in clinical trials of immune-checkpoint inhibitors in hepatocellular carcinoma.

Biomarker	Cut-off	Agent	Significant Association	Findings	Study Name (reference)	Phase
PD-L1 expression	CPS ≥1	Pembrolizumab	Better ORR and PFS	PD-L1 expression was correlated with ORR and PFS	KEYNOTE-224 (15)	II
	TPS ≥1	Pembrolizumab	No	No correlation between PD-L1 expression and ORR or PFS	KEYNOTE-224 (15)	II
	TPS ≥1	Nivolumab	Better OS	Median OS: 28.1 *vs.* 16.6 months	CheckMate 040 (29)	I/II
	TPS ≥1	Nivolumab	No	ORR: 29% *vs.* 20% (ITT population) ORR: 13% *vs.* 22% (Asian cohort)	CheckMate 040 (Asian cohort) (18)	I/II
	NA	Nivolumab *vs.* sorafenib	Better ORR	ORR: 28% *vs.* 12%	CheckMate 459 (17)	III
	NA	Pembrolizumab	No	No correlation between PD-L1 expression and ORR	NCT02658019 (21)	II
Four-gene signature	/	Nivolumab	Better OS	Four-gene inflammatory signature (*CD274*, *CD8A*, *LAG3*, and *STAT1*) was associated with improved OS	CheckMate 040 (29)	I/II
Plasma TGF-β	TGF-β <200 pg/mL	Pembrolizumab	Better OS and PFS	Median OS: >25 months *vs.* 7 months; median PFS: >25 months *vs.* 2 months	NCT02658019 (21)	II
NLR or PLR	/	Nivolumab	Better OS	OS was longer in patients with NLR or PLR in the lower tertile	CheckMate 040 (29)	I/II
Serum AFP	AFP <400 µg/L	Nivolumab	Better OS	Median OS: 16.8 *vs.* 13 months	CheckMate 040 (29)	I/II

CPS, combined positive score; TPS, tumor proportion score; ORR, overall response rate; PFS, progression-free survival; ITT, intent-to-treat; NA, not available; OS, overall survival; NLR, neutrophil-to-lymphocyte ratio; PLR, platelet-to-lymphocyte ratio.

The contradictory results of these clinical trials may be partly due to limitations in the detection of PD-L1 levels, including a lack of standard methods for evaluating PD-L1 expression and its spatial and temporal heterogeneity, as well as the absence of standard thresholds that allow the determination of “overexpression” (30, 31). The expression of PD-L1 is measured using immunohistochemistry on formalin-fixed paraffin-embedded (FFPA) sections; however, two methods are used for defining PD-L1-positive expression, namely, the ratio of PD-L1-positive tumor cells—the so-called tumor proportion score (TPS)—and the ratio of PD-L1-stained tumor and immune cells, the so-called combined positive score (CPS). The KEYNOTE-224 phase II trial evaluated PD-L1 using both scoring methods, with the CPS turning out to be a more applicable biomarker (15). In addition, the predictive value of PD-L1 expression may be underestimated as it is commonly evaluated at a single time point, even though PD-L1 expression is dynamic and inducible (32, 33). PD-L1 is heavily glycosylated and such modifications significantly affect the detection performance and therapeutic efficacy of PD-L1 antibodies (34, 35). A method was developed to resolve this issue that involved removing the glycan moieties from cell-surface antigens *via* enzymatic digestion, which boosted the positivity rate of PD-L1 detection in tumor samples (35).

### Tumor Mutational Burden/Microsatellite Instability

Neoantigens, arising as a consequence of tumor-specific mutations, are hypothesized to generate robust immune responses (36). The tumor mutational burden (TMB) refers to the number of nonsynonymous mutations found in the genome of a single tumor, including alterations in DNA damage response genes and those encoding the DNA polymerase epsilon (POLE) and delta (POLD) catalytic subunits, and has been assessed for its potential as a biomarker in multiple tumor types (37, 38). It is thought that tumors with a greater TMB can produce a greater number of neoantigens. One cross-cancer study reported that tumors with a high TMB are positively correlated with responses to anti-PD-1/PD-L1 therapy, and a greater TMB is also associated with higher PD-L1 expression in tumor cells (39). To examine this association more broadly, another study analyzed the clinical and genomic data of thousands of patients with advanced cancer and identified an association between a higher TMB and improved survival linked to ICI treatment in most of the cancer types assessed (40). Several studies have shown that a higher TMB is associated with immune microenvironment diversification and worse prognosis in HCC patients (41, 42). However, its putative role as a predictive biomarker has not been reported in HCC patients treated with either nivolumab or pembrolizumab, and whether the TMB has value as a predictive marker for ICI efficacy remains unclear.

A study evaluated the frequency of genomic biomarkers of ICI response in 755 HCC patients and found that the median TMB was four mutations/Mb, with only six tumors (0.8%) found to be TMB-high (43). Furthermore, in a small case series (*N*=17), the TMB showed no correlation with ICI response. As in the case of PD-L1 expression, however, TMB determination lacks standardized thresholds and there is variability in quantification methods (44). Thus, the clinical value of TMB should be interpreted with caution. Recently, Wong et al. evaluated the TMB in 29 HCC patients through targeted next-generation sequencing (tNGS) on both fresh and archival samples. The authors reported that, while fresh HCC samples were better sources of tumor DNA (45), the low median TMB values observed may limit the usefulness of the TMB as a predictor of response to immunotherapy in HCC.

DNA mismatch repair (MMR) represents a key mechanism for the maintenance of genomic integrity and stability. A deficiency in MMR activity results in a hypermutator phenotype known as microsatellite instability (MSI) (46). High mutation rates in somatic cells are believed to amplify the neoantigen load, leading to lymphocyte activation and enhanced cancer susceptibility to immunotherapy (47, 48). A study assessed the efficacy of PD-1 blockade with pembrolizumab monotherapy in 86 patients with advanced MMR-deficient cancers across 12 different cancer types. Based on ORRs and complete response (CR) rates of 53% and 21%, respectively, the results of this study support that MSI may serve as a predictor of the clinical response of solid tumors to PD-1 blockade (49). Although MSI is considered useful as an agnostic histological indicator for the selection of responders to ICI therapy, there is nonetheless a lack of data for HCC patients. Ang et al. assessed 542 HCC specimens for MSI and found that only one (0.2%) was MSI-high and TMB-high (43), while several other studies have reported that the prevalence of MSI in HCC is also generally low (50, 51). These findings highlight that MSI may not be an ideal biomarker to predict responses to ICIs in HCC patients.

The low frequency of high TMB and high MSI limits their exploration as predictive biomarkers in HCC, while the number of cases included in existing studies is also relatively low (44). However, given that MSI has shown good predictability in immunotherapy for other types of cancers, research efforts should continue to focus on exploring its predictive potential in HCC, including combining data from multiple clinical trials to obtain a sufficiently large sample size that would allow the evaluation of its role in HCC-targeted immunotherapy.

### Tumor-Infiltrating Lymphocytes

Because T-cell infiltration within the TME is a prerequisite for immune checkpoint blocking (52), baseline intratumoral T-cell density and phenotype have been extensively studied and closely connected with ICI responses in melanoma and other tumors (53, 54). A retrospective biomarker analysis undertaken in the CheckMate 040 trial indicated that, although not statistically significant, increased numbers of CD3+ or CD8+ tumor-infiltrating T cells were correlated with a trend towards improved OS in nivolumab-treated HCC patients (29). This study further indicated that an association existed between an increased frequency of CD3+ T cells and the best overall response. A different study reported that an increase in CD8+ T cells in six-week tumor biopsies was connected with clinical benefits for HCC patients who received tremelimumab plus ablation combination therapy (55). Furthermore, Kaseb et al. found that the clinical response of HCC patients who received perioperative immunotherapy (nivolumab plus ipilimumab) followed by surgical resection was correlated with an increase in CD8+ T-cell infiltration, and, specifically, with two effector T-cell clusters (CD3+CD8+CD45RO+Eomes+ and CD3+CD8+CD45RO+Eomes+CD57+CD38low) (56).

The connection between TILs and ICI treatment response has also been investigated in other immune cell types. Ng et al. analyzed 49 HCC samples from patients treated with ICIs and reported that patients with a high intratumoral CD38+CD68+ macrophage density had a better median OS compared with those with low CD38+CD68+ macrophage density (34.43 *vs.* 9.66 months) (57), likely because CD38hi macrophages produce greater amounts of interferon-gamma (IFN-γ) and related cytokines. Notably, some of these 49 patients had received combination immunotherapy. The influence of immune cell infiltration on the effectiveness of ICIs has also been studied in multifocal HCC, with the results showing that small nodules are more sensitive to anti-PD-1 therapy than large nodules, while small tumors exhibit greater immune cell infiltration and an upregulated interferon signature compared with large tumors (58).

Deep single-cell RNA sequencing was performed on 5,063 T cells isolated from peripheral blood, tumor tissue, and adjacent normal tissues of six HCC patients (59). The results demonstrated that specific subsets, such as exhausted CD8+ T cells and regulatory T cells (Tregs), were preferentially enriched in HCC. The authors further identified that increased expression of layilin (encoded by *LAYN*) was associated with tumor-infiltrating Tregs and activated CD8+ T cells. While these findings need to be validated in a larger cohort, they will undoubtedly open a new avenue for further research into the potential of utilizing CD8+ T-cell infiltration as a biomarker in HCC.

Although it is important to focus on lymphocytes in the TME, applying them as biomarkers in the clinic remains a distant prospect. How to select lymphocytes with specific characteristics, how to define the positive criteria are among the many unanswered questions. Joint evaluation of the number of multiple immune cells and PD-L1 expression to develop TME scores may be one of the answers.

### Specific Gene Alterations

Over recent years, molecular profiling associated with the advent of NGS has provided information on actionable targets and identified specific gene alterations associated with responses to ICIs (60).

Mutations in the *CTNNB1* gene, which lead to the activation of the WNT/β-catenin signaling pathway, are characteristic of immune-excluded HCC types (cold tumors) and are associated with significantly lower enrichment scores for several immune signatures (61, 62). Harding et al. employed NGS to determine which type of patient with advanced HCC might benefit from systemic treatments (63). For a subgroup of 31 patients treated with diverse ICIs, activating alterations in the WNT/β-catenin pathway were associated with lower disease control rates (DCRs) (0% *vs.* 53%), shorter median PFS (2.0 *vs.* 7.4 months), and shorter median OS (9.1 *vs.* 15.2 months) compared with those in WNT wild-type HCC. Additionally, β-catenin-driven tumors were reported to be resistant to PD-1 therapy in a mouse model of HCC, while the expression of chemokine (C-C motif) ligand 5 (CCL5) could restore the β-catenin-associated loss of immune surveillance (64).

In addition, *TP53* gene alterations were the most frequently identified mutations in HCC patients and were mutually exclusive with *CTNNB1* mutations (65). Studies have shown that *TP53* alterations are strongly related to the immune microenvironment in HCC, with less CD8+ T cell infiltration and more FOXP3+ Treg infiltration, resulting in the downregulation of the immune response (66–68). *TP53* dysfunction was shown to be linked to chromosomal instability (defined as high broad copy-number alteration loads) and immune-excluded traits in HCC (69). Conversely, Yang et al. reported that HCC patients carrying TP53 neoantigens showed higher cytotoxic lymphocyte infiltration and longer OS (70).

Recent studies have indicated that immune-related long noncoding RNAs (lncRNAs) may predict ICI treatment responses in HCC. Peng et al. reported that the host gene of lncRNA *MIR155* was strongly positively correlated with the expression of CTLA-4 and PD-L1 in HCC tissues, and showed predictive value for the curative effect of ICI therapy (71). Additionally, Zhang et al. identified an immune-related lncRNA signature that correlated with worse survival and was an independent prognostic biomarker for HCC patients (72). That this signature was associated with immune cell infiltration and ICI treatment-related molecules (including PD-L1, PD-L2, and IDO1) suggests that it may have the potential to measure the response to ICI immunotherapy. Several studies have reported on the ability of immune-related lncRNAs to predict prognosis in HCC patients (73–76). However, prospective validation in HCC patients who received ICI treatment is still lacking. MicroRNAs and circular RNAs have also been reported to reshape the immune microenvironment in HCC (77, 78). Mo et al. identified 5-methylcytosine (5mC)-associated molecular subtypes in HCC and found that they were associated with responses to immunotherapy (79).

Several studies have indicated that cancer cell-intrinsic epigenetic alterations are associated with carcinogenesis and tumor progression (80–83), as well as with changes in the TME, such as infiltration of tumor-associated lymphocytes and expression of immune checkpoint molecules (84, 85), indicative of their potential as predictors of immunotherapy. Furthermore, given that DNA methylation can be measured in liquid biopsies, epigenetic biomarkers may provide additional advantages, such as low patient invasiveness (86). In patients with melanoma receiving anti-PD-1 immunotherapy, the hypermethylation of the T-cell costimulatory receptor TNFRSF9 was reported to be correlated with poor PFS and treatment response (87). In a multicenter study, Duruisseaux et al. established an epigenomic profile based on a microarray DNA methylation signature (EPIMMUNE) in a discovery set of tumor samples from patients with advanced nonsmall cell lung cancer (NSCLC) who had received anti-PD-1 therapy (88). The authors found that the unmethylated status of the T-cell differentiation factor forkhead box P1 (FOXP1) was associated with improved PFS and OS, and, therefore, possessed good predictive value for the efficacy of anti-PD-1 treatment. Another study identified a broad DNA methylation signature in peripheral blood mononuclear cells and T cells of HCC patients that differed from that of non-HCC patients (89). Additionally, differences in immune infiltrates related to the methylation levels of cell division cycle-associated (CDCA) family genes in HCC were reported to have potential as predictive biomarkers for responses to immunotherapy (90). Notably, Llopiz et al. reported that an epigenetic drug/ICI combination exerted synergistic antitumor effects in a murine model of HCC (91), thereby providing further evidence that DNA methylation signatures may be related to the efficacy of immunotherapy, and also have the potential to serve as biomarkers of combination therapy.

Combined, these observations indicate that the presence of specific gene alternations, especially those related to *CTNNB1*, *TP53*, noncoding RNAs, and methylation, may influence the response to ICI treatment through interacting with the immune microenvironment. Although these studies are still preliminary, such alterations could represent novel biomarkers for HCC patient selection, and patient exclusion in particular.

### Immune-Related Gene Signatures

Comprehensive analyses of tumor transcriptomic profiling data have recently been conducted to characterize the responsiveness of the immune microenvironment to ICI treatment. Ayers et al. identified an 18-gene T-cell-inflamed expression profile and a 6-gene IFN-γ signature that could predict responses to pembrolizumab therapy across multiple solid tumors (92). Based on gene expression profiles, Sia et al. classified 25% of HCCs as an “immune-specific class” characterized by high expression levels of PD-L1 and markers of cytolytic activity. This class further comprised two subtypes, namely, an active immune subtype, characterized by significant enrichment of T cells and IFN signatures, and an exhausted immune subtype, associated with a T-cell exhaustion signature and immunosuppressive components (TGF-β and M2 macrophages) (62).

The previously-mentioned retrospective analysis of the findings of the CheckMate 040 trial also included the evaluation of the predictive value of a four-gene inflammatory signature (*CD274* [PD-L1], *CD8A*, *LAG3*, and *STAT1*). This signature was found to be associated with improved response and OS related to nivolumab therapy, both in the dose-escalation and dose-expansion phases (29), and may be indicative of IFN-γ/STAT1-dependent CD8+ T-cell expansion, LAG-3-dependent T-cell exhaustion, and/or an immune-suppressed TME with high PD-L1 expression. Importantly, however, although the differences in the above-mentioned clinical trial data were reported to be statistically significant, this gene signature does not adequately distinguish between responders and nonresponders. Recently, a five-gene immune-related signature (*LDHA*, *PPAT*, *BFSP1*, *NR0B1*, and *PFKFB4*) was identified and used to establish a prognostic model for responses to HCC treatment that could stratify patients who were sensitive to immunotherapy (93). Other studies using different gene combination strategies have also reported their potential to predict ICI responses by reflecting the characteristics of the immune microenvironment (94, 95). Nevertheless, these signatures need further testing for clinical application.

Immune-related gene signatures are associated with the same obstacles as TILs, namely, how to select the best gene combinations and positive cut-off values. A feasible strategy involves undertaking a wide-ranging bioinformatics analysis of the existing databases to identify possible gene combinations and then verify them through basic research and clinical trials.

### Biomarkers in Peripheral Blood

The continuous availability of tumor samples from ICI-treated patients is crucial for biomarker research; however, this is difficult to achieve owing to the invasive nature of biopsies. In contrast, circulating biomarkers can be easily collected and repeatedly measured after treatment, rendering them more convenient for use in the clinic.

Studies have shown that TGF-β plays a central role in immune suppression within the TME and tumor immune evasion (96). In HCC, the potent immune inhibitory function of Tregs is a major obstacle to generating an effective antitumor response, and Treg activation is modulated by the TGF-β pathway (97). In addition, TGF-β enables tumor evasion from host immune responses in part through enhancing SMAD3-mediated PD-1 expression on TILs (98). The TGF-β signaling pathway is activated at the transcriptional level in most HCCs (99, 100). A strong association was identified between the TGF-β signature and the exhausted immune signature in HCC (62, 99). In a phase II trial, several representative circulating biomarkers were analyzed in 29 patients with unresectable HCC treated with pembrolizumab, with the results showing that baseline plasma TGF-β levels of <200 pg/mL were an effective predictor of better OS and PFS (21). The therapeutic co-administration of TGF-β-blocking and anti-PD-L1 antibodies was reported to facilitate T-cell penetration into the center of tumors and elicit strong antitumor immunity (101). Clinical trials of the TGF-β receptor I inhibitor galunisertib have been conducted, and have reported median OS durations of 16.8 months in patients with advanced HCC with baseline AFP <1.5 × ULN (102). Briefly, TGF-β may serve as a negative predictive biomarker for ICI therapy given that HCCs with strong TGF-β and exhausted immune signatures may be resistant to PD-1 blockade, while HCC patients with an activated TGF-β signature are expected to benefit from a combination of ICI and TGF-β inhibitor therapy (99). Thus, it seems likely that drugs targeting both TGF-β and PD-1/PD-L1, such as bintrafusp alfa, will play a role in the treatment of HCC in the future.

Several soluble immune checkpoint-related proteins were recently shown to have promising predictive value in various cancer types. Chen et al. reported that metastatic melanomas released exosomes carrying PD-L1 that suppressed CD8+ T-cell function and supported tumor growth. Additionally, the authors reported that the magnitude of the increase in the levels of circulating exosomal PD-L1 during the early stages of treatment could discriminate clinical responders from nonresponders (103). One preclinical study using mouse models indicated that the removal of exosomal PD-L1 inhibits tumor growth, even in models resistant to PD-L1 blockade (104).

Soluble PD-L1 (sPD-L1) has been reported to be correlated with responses to immunotherapy in patients with NSCLC (105). Additionally, several studies have focused on the prognostic value of sPD-L1 and identified high serum sPD-L1 levels as an independent predictive factor for poor outcomes in HCC patients (106–108). Whether an association exists between sPD-L1 and intratumoral PD-L1 expression levels remains unclear, with contradictory results having been reported (106, 108). Moreover, there is a lack of data on the ability of exosomal PD-L1 or sPD-L1 to predict clinical outcomes in HCC patients following ICI therapy, which warrants further study.

Circulating immune cells in peripheral blood have been extensively evaluated as predictive biomarkers (105, 109). Agdashian et al. tested the combination of anti-CTLA-4 treatment (tremelimumab) with locoregional therapy in HCC patients and found that the frequency of CD4+PD-1+ cells among peripheral blood mononuclear cells at baseline was higher in patients responding to therapy than in nonresponding patients (110). In addition, low baseline peripheral B cell PD-1 positivity and constant posttreatment monocyte PD-L1 positivity were observed to be associated with disease control in 16 HCC patients treated with nivolumab (111). Dharmapuri et al. evaluated the relationship between the neutrophil–lymphocyte (NLR) and platelet–lymphocyte (PLR) ratios and survival outcomes in HCC patients treated with nivolumab and reported that patients who achieved a partial or CR had significantly lower posttreatment NLRs and PLRs (112). The predictive value of the NLR and the PLR was also indicated in the CheckMate 040 trial (29).

Necrotic or apoptotic tumor cells release DNA carrying tumor-related genetic and epigenetic alterations into the bloodstream, thereby helping to overcome the limitations related to sample availability. Circulating tumor DNA (ctDNA) can reportedly predict tumor responses to ICI therapy in several types of tumors; specifically, it can be used to distinguish pseudoprogression from true progression (113–115). However, a study analyzing the mutational landscape of advanced HCC using ctDNA reported that WNT pathway-related mutations were not associated with clinical outcomes after ICI therapy (65), highlighting that further investigation is needed to determine whether ctDNA can indeed serve as a predictive biomarker in HCC. Meanwhile, although the blood TMB was reported to have good predictive value in some types of cancer (116, 117), evidence is lacking for HCC. Winograd et al. sought to detect PD-L1-expressing circulating tumor cells (CTCs) in HCC patients and found that there was a strong association between the presence of PD-L1+ CTCs and favorable treatment responses to PD-1 blockade (118).

High AFP levels are considered to be a prognostic marker for poor clinical outcomes in HCC patients. Recently, a posttreatment decline in serum AFP levels was reported to be associated with higher ICI treatment efficacy in advanced HCC (119, 120). In addition, Spahn et al. reported that baseline levels of AFP of <400 µg/L at the start of ICI treatment were associated with higher rates of CR or partial response (PR) as best responses (121). However, the results of the checkmate040 clinical trial showed that although baseline AFP <400 µg/L was associated with longer OS compared with AFP ≥400 µg/L, the ORR and DCR were similar regardless of baseline AFP levels (29). Given that the AFP level is closely related to the baseline characteristics of patients, its predictive effect should be interpreted with caution.

Biomarkers in peripheral blood have the great advantage of low invasiveness, while a large number of studies have also shown their potential as predictors of immunotherapeutic efficacy. However, supporting data from large-sample clinical trials are still lacking. In addition, there is currently no evidence that the predictive accuracy of blood samples is better than that of tissue samples for any given biomarker. Nevertheless, these studies provide insights for future research into biomarkers for HCC immunotherapy.

### Gut Microbiota

Increasing evidence has indicated that the gut microbiota plays a crucial role in the development and regulation of innate and adaptive immunity, while several studies have described its value in predicting the efficacy of ICIs. For instance, analysis of baseline gut microbiota composition of fecal samples from patients with melanoma or NSCLC before immunotherapy treatment has indicated that commensal microbial composition is associated with an ICI response (122, 123).

A meta-analysis undertaken on 2,424 samples through 16S RNA gene sequencing and machine based-learning indicated that among several major dysbiosis-related diseases, liver cirrhosis is the condition where changes in the gut microbiome most accurately predict the presence of disease (124). Given that there is an anatomical connection between the liver and the gut, and that HCC occurs in the context of chronic liver inflammation concomitant with a defective intestinal barrier and increased hepatic exposure to bacterial products, it seems likely that a relationship exists between the gut microbiota and responses to ICI therapy. Indeed, increasing evidence points towards a key role of the bacterial microbiome in promoting the development of HCC (125). In the context of chronic inflammation, intestinal bacterial translocation is detected by Toll-like receptor (TLR) 4 present on resident liver cells through its ligand lipopolysaccharide, which leads to the upregulation of the expression of the hepatomitogen epiregulin and, consequently, the promotion of hepatocarcinogenesis (126). Microbiota-derived metabolites can also affect the development of HCC. For instance, the gut microbial metabolite deoxycholic acid acts in concert with lipoteichoic acid to enhance the tumor-promoting phenotype of hepatic stellate cells and promote the expression of COX2 through TLR2, resulting in the suppression of antitumor immunity (127). Additionally, gut microbial-dependent bile acid metabolism modulates liver tumor growth by regulating the hepatic expression of CXCL16, a mediator of natural killer T (NKT) cell recruitment (128). Furthermore, Arpaia et al. found that microbe-derived short-chain fatty acids facilitate extrathymic Treg generation (129). Studies have also shown that the gut microbiota may play an important role in regulating ICI treatment responses (130, 131). For instance, fecal microbiota transplantation from cancer patients who responded to ICIs into sterile or antibiotic-treated mice improved the response to anti-PD-1 therapy (122, 123). Moreover, Zheng and colleagues recently reported the dynamic variation in the composition of the gut microbiome during anti-PD-1 immunotherapy in HCC by metagenomic sequencing (132). They observed that fecal samples from patients responding to immunotherapy showed higher taxonomic richness and greater gene counts compared with those of nonresponders; microbial composition remained relatively stable in the responder group, whereas in nonresponders, *Proteobacteria* abundance markedly increased from week 3 and became predominant at week 12. In addition, antibiotic administration at the initiation of ICI treatment was reportedly associated with worse outcomes, indicative of the influence of the gut microbiota on HCC treatment (121). A clinical trial (NCT03785210) to evaluate the effects of combined antibiotic (vancomycin) and ICI therapies is currently underway.

However, in the aforementioned studies, there was no overlap in gut microbiota associated with responses, which may be due to differences in etiology, geographic location, nutritional intake, and techniques used to analyze the samples. Moreover, Rosshart et al. found that the gut microbiome of laboratory mice differs significantly from that of closely related species in the wild (133), implying that caution is needed when generalizing these research results. The gut microbiota can be influenced by many environmental, dietary, and lifestyle factors, all of which can potentially affect the immune system and, consequentially, regulate the response to ICIs (134). Given these complications, the application of gut microbiota as a biomarker for clinical use remains a distant possibility.

### Others

The T-cell receptor (TCR) is composed of multiple antigen-specific peptide chains. Recent studies have used high-throughput sequencing for the in-depth elucidation of the composition and distribution of the TCR. There have been several attempts to use the TCR as a predictive biomarker for ICI responses. A more clonal TCR repertoire or oligoclonal TIL expansion is associated with a better response to PD-1 blockade in melanoma patients (135, 136). In addition, baseline TCR diversity in peripheral blood has been associated with clinical outcomes following ipilimumab treatment in metastatic melanoma (137). Despite the lack of data regarding the suitability of employing TCR as a biomarker in HCC, its predictive potential nevertheless warrants further investigation given the novel TCR analysis approaches proposed (138, 139). Lin et al. found that the combination of TCR repertoires and TNM stage could serve as an efficient prognostic indicator in patients with HBV-associated HCC (140). Additionally, Han et al. identified several specific *TRBV*–*TRBJ* combinations that could distinguish the TCR repertoires of HCC patients from those of healthy adults and thus have the potential to serve as novel biomarkers (141).

Pfister et al. conducted a meta-analysis that incorporated more than 1,600 patients with advanced HCC in three randomized phase III clinical trials and reported that PD-L1 or PD-1 inhibitors did not improve survival in patients with nonviral HCC, particularly NAFLD (142). This was likely due to the progressive accumulation of exhausted, unconventionally activated CD8+PD-1+ T cells, which contributed to inducing NAFLD/HCC rather than carrying out or enhancing immune surveillance. However, in the CheckMate 040 phase I/II trial, which enrolled HCC patients with or without HBV or HCV infection, responses to nivolumab were observed irrespective of HCC etiology (14).

Epithelial-to-mesenchymal transition (EMT) has been implicated as a resistance mechanism that helps to promote the immune evasion of cancer cells (143). High expression of PD­L1 in HCC patients is reported to be associated with an EMT phenotype and be a predictor of poor survival (41). The correlation between PD-L1 expression and EMT presents a theoretical foundation to investigate EMT as a negative biomarker for ICI responses.

Matrix metalloproteinase 9 (MMP-9) secreted by tumor-associated macrophages was recently reported to be a potential predictor of immune characteristics and immunotherapeutic responses in HCC (144, 145). One study measured *ADAM9* mRNA levels in blood samples derived from patients with advanced HCC. Among four patients treated with nivolumab therapy, two who exhibited a clinical response also showed significant decreases in serum *ADAM9* mRNA levels, whereas the two who displayed no response to nivolumab did not. Although the sample size was small, the results of this study nevertheless suggested that *ADAM9* mRNA might serve as a predictive biomarker for clinical responses (146).

In addition, Qayyum et al. undertook an interesting prospective study that included 15 patients with advanced HCC treated with pembrolizumab and found that the changes in HCC stiffness as measured by magnetic resonance elastography (MRE) at 6 weeks was significantly associated with OS (147). This was the first proposed imaging-based predictor of immunotherapy outcome in HCC and opens up new avenues for predictor selection.

### Potential Biomarkers for irAEs

Between 15% and 25% of ICI-treated HCC patients undergo grade 3/4 treatment for immune-related adverse events, including fatigue, pruritus, rash, diarrhea, and increases in aspartate aminotransferase and alanine aminotransferase levels (14, 15). Although ICI treatment-related adverse events are manageable and less frequent than those seen with chemotherapy, it is still necessary to identify biomarkers that can predict irAEs to alleviate unnecessary suffering in patients.

Most studies have focused on identifying biomarkers for predicting the efficacy of immunotherapy, while relatively few studies have investigated biomarkers relating to irAEs. Moreover, a large proportion of research findings come from patients with melanoma (148). Nevertheless, these studies can serve as a reference for identifying biomarkers that can predict irAEs in HCC. Baseline serum IL-6 and IL-17 levels were significantly associated with an increased risk of severe toxicity in patients treated with ipilimumab (149, 150), with IL-17 being related to severe diarrhea/colitis. A retrospective review involving 167 adult patients with solid tumors indicated that increased baseline lymphocyte counts are associated with a greater risk for irAEs in patients treated with nivolumab or pembrolizumab (151). The detection of autoantibodies has been suggested to predict the development of irAEs related to the autoantibodies, and two studies evaluating antithyroid antibodies and diabetes-related autoantibodies have been reported (152, 153). Baseline gut microbiota enriched in *Faecalibacterium* spp. and other members of the Firmicutes is associated with a more frequent occurrence of ipilimumab-induced colitis (154).

Rogado et al. reported that ICI treatment was markedly more beneficial for patients with advanced cancer presenting with irAEs than for those without irAEs (ORR: 82.5% *vs.* 16.6%; PFS: 10 *vs.* 3 months) (155). Cutaneous or early irAEs are associated with improved survival in melanoma patients treated with nivolumab (156, 157). Future studies should address this association to explore the underlying biological mechanisms related to ICI efficacy, while how to balance the incidence of irAEs and the immunotherapeutic response also merits serious consideration.

## Conclusion

In HCC, although several studies have been conducted to identify predictive biomarkers that would allow the stratification of patients who could benefit from ICI treatment, few have been prospectively validated and none have resulted in the rewriting of the current clinical guidelines or entered into clinical practice. Here, we summarized the progress of immunotherapy for HCC over recent years, with a particular emphasis on predictive biomarkers. However, as HCC-related immunotherapy is still in its infancy, basic research and clinical trials exploring the predictive efficacy of immunotherapy biomarkers are still limited, and it is not yet possible to determine which biomarker(s) can effectively predict the efficacy of immunotherapy. It is particularly noteworthy that biomarkers represent continuums and undergo dynamic changes in a population of patients. Accordingly, it is pivotal to obtain samples from patients both before and during treatment to evaluate these dynamic changes and properly determine the predictive value of the assessed biomarkers, while adequate consideration should be given to their application in clinical practice. Furthermore, we only reviewed the biomarkers that predict responses to immune monotherapy, especially anti-PD-L1 and anti-PD-1 agents. However, given the success of the IMbrave150 phase III clinical trial, it is clear that antivascular therapy combined with immunotherapy has great potential in patients with advanced HCC, and combination therapy may be the direction of cancer treatment in the future. But while combination therapy can prolong the OS of HCC patients, it also complicates the patient selection process. The development of research techniques such as NGS, single-cell RNA sequencing, and artificial intelligence should allow for a more comprehensive understanding of the various components of the TME and their interactions, and potential biomarkers could be widely screened on a genomic scale to identify the predictors of treatment efficacy.

## Author Contributions

PZ conceived of the topic for this review. All authors contributed to the article and approved the submitted version.

## Funding

This work was funded by the National Natural Science Foundation of China (No. 81974483).

## Conflict of Interest

The authors declare that the research was conducted in the absence of any commercial or financial relationships that could be construed as a potential conflict of interest.

## Publisher’s Note

All claims expressed in this article are solely those of the authors and do not necessarily represent those of their affiliated organizations, or those of the publisher, the editors and the reviewers. Any product that may be evaluated in this article, or claim that may be made by its manufacturer, is not guaranteed or endorsed by the publisher.
